# Machine Learning: New Potential for Local and Regional Deep-Seated Landslide Nowcasting

**DOI:** 10.3390/s20051425

**Published:** 2020-03-05

**Authors:** Adriaan L. van Natijne, Roderik C. Lindenbergh, Thom A. Bogaard

**Affiliations:** 1 Department of Geoscience and Remote Sensing, Delft University of Technology, P.O. Box 5048, 2600 GA Delft, The Netherlands; R.C.Lindenbergh@tudelft.nl; 2 Department of Water Management, Delft University of Technology, 2600 GA Delft, The Netherlands; T.A.Bogaard@tudelft.nl

**Keywords:** deep-seated landslide, machine learning, remote sensing, early warning systems, hazard assessment

## Abstract

Nowcasting and early warning systems for landslide hazards have been implemented mostly at the slope or catchment scale. These systems are often difficult to implement at regional scale or in remote areas. Machine Learning and satellite remote sensing products offer new opportunities for both local and regional monitoring of deep-seated landslide deformation and associated processes. Here, we list the key variables of the landslide process and the associated satellite remote sensing products, as well as the available machine learning algorithms and their current use in the field. Furthermore, we discuss both the challenges for the integration in an early warning system, and the risks and opportunities arising from the limited physical constraints in machine learning. This review shows that data products and algorithms are available, and that the technology is ready to be tested for regional applications.

## 1. Introduction

Landslides are a major hazard to human life and society, killing over 55,000 people over the period of 2004–2016 [1], and causing an estimated average economic loss of €4.7 billion per year in Europe alone [2]. To protect the public, landslides have been a major research topic for the last few decades, strengthened by recent commitments such as the Sendai agreement for disaster risk reduction and the ‘Kyoto 2020 commitment’ to reduce landslide disaster risk [3,4].

New data and data integration methods offer new possibilities for landslide forecasting, especially for slow-moving, deep-seated landslides. Here, we provide a perspective on the possibilities, applications, and challenges of both local and regional deformation nowcasting and its inclusion in early warning systems. A nowcast describes the current, estimated system state, and provides an outlook on the coming days.

We focus on slow moving, deep-seated landslides on natural slopes, for which deformation is controlled by hydro-meteorological conditions. These landslides are characterized by gradual, non-catastrophic deformations of millimeters to decimeters per year and can be monitored and modeled over at least multiple years. They are considered to be in a state of so-called limit-equilibrium undergoing continuous deformation, but may accelerate or stabilize when conditions change [5].

Landslide geologists have compiled local, spatial landslide susceptibility maps since the 1970s [6,7,8]. Such maps delineate landslide-prone areas based on historic landslides and expert analysis of landscape properties. However, susceptibility maps only indicate where a landslide may occur without a specific time frame [9]. Advances in geospatial data acquisition and processing in the last 50 years have greatly influenced the field. Current, quantitative, regional [10,11] and global [12,13] susceptibility maps are based on statistics rather than expert judgement alone and use historical landslide events for calibration/training.

Extensive reviews have been written on the state-of-the-art in susceptibility mapping, either summarising the methods available or making quantitative analyses of the classification process [14,15,16,17,18,19,20,21,22]. However, whereas static susceptibility maps have proven their value for spatial planning, when monitoring landslide stability, both for single landslides as well as on a regional scale, time dependency cannot be neglected [23].

In practice, most shallow landslides are triggered by extreme precipitation events [24], or by a combination of hydro-meteorological events. However, failure modes for fast-moving landslides are not applicable to deep-seated landslides and statistical relationships—for example, the intensity-duration thresholds for precipitation do not offer sufficient predictive power [25]. Although seismic events can not be neglected as a trigger, earthquakes are a different triggering mechanism that is explicitly not considered here. Moreover, earthquakes with a magnitude lower than 4.0–5.5 are less likely to trigger a landslide [26,27].

Sliding behaviour is governed by the balance of forces within the landslide, that is, the relation of the shear strength of the soil to the shear (sliding) force applied by the gravitational forces acting on the landmass. Changes in hydrology change the balance between these forces [28]. Therefore, infiltration of rain, or delayed infiltration from snow melt, and the subsequent rise of the pore water pressure shift the balance of forces as the increased pore pressure weakens the soil.

As a major result of the landslide process, displacement is a key parameter to capture the interaction between landslide deformation and hydro-meteorological conditions—the relation between soil moisture and increased deformation has been observed in the field [29,30]. Studies focused on the progressive deformation of individual landslides have appeared in recent years, connecting deformation to the conditions on the slope (e.g., [31], Table 4). These studies claim good results in predicting landslide deformation based on hydro-meteorological conditions using machine learning algorithms and limited geomechanical modelling.

Machine learning offers new possibilities to bypass the microscale physics of the landslide, estimating the behaviour based on large data sets of previous responses to hydro-meteorological conditions as an intermediate step between passive monitoring and extensive (numerical) modelling of the landslide. This is done either by incorporating physics, such as the groundwater level, in the statistical model [32,33] or by estimating the deformation rate of the landslide directly based on the hydro-meteorological time series. Although there is no strict link between data availability and predictive capacity [34], such an approach has been proven to work at the landslide scale, such as by the examples discussed in Section 3.

At the advent of both local and regional landslide nowcasting, data availability is more important than ever, especially data that can be used for training of new machine learning algorithms. Their spatial properties and significance in the landslide process, as well as their temporal availability and suitability for automated data integration should be taken into account.

In this paper, we highlight the opportunities of machine learning using static and dynamic remotely sensed data sources for monitoring and nowcasting of deep-seated landslides. The overarching aim is to arrive at a near real-time, machine learning-based, local and regional early warning system for precipitation-initiated acceleration of slowly deforming slopes. Hereto, we will discuss conditional data sources, and dynamic causal and triggering factors. Then, we will discuss various machine learning algorithms from landslide literature. The paper finally discusses the current limitations and challenges, as well as the potential of combining local near real-time ground sensed data with the remotely sensed data.

## 2. Monitoring Opportunities for Slow-Moving Deep-Seated Landslides

Landslides are “the movement of a mass of rock, debris, or earth (soil) down a slope” [9]. Pre-disposing factors are essential for a landslide to form and have been integrated in susceptibility maps in the past. However, the landscape is stable most of the time. Leading up to a landslide are causal and triggering factors—these first allow the landslide to happen, making it sensitive to triggering factors that initiate the movement.

The deformation of the landslide is an indicator of stress at the sliding plane, which is often impossible to measure directly. For landslides in limit-equilibrium, the balance of forces can be approximated by the Mohr–Coulomb failure criterion [35] under the assumption of a single sliding plane. Moreover, past landslide events are indicative of future behaviour, and similar landslides will exert similar behaviour in similar situations [9,36]. Furthermore, the behaviour of continuous, slow-moving, non-catastrophic landslides can be followed over longer periods of time [14].

Working from the various reviews of susceptibility maps [15,17], a list of key variables that can be acquired from satellite observations is shown in Table 1. Slope, and related properties of aspect and curvature, are typically identified as primary conditioning factors for landslides: no slope, no landslide. In most susceptibility analyses, dynamic triggering factors like precipitation or snow melt are not included, but should be included in a landslide nowcasting solution. Toe erosion—either gradual by water or sudden by building activity—is often captured in land use. However, local situations may require extra variables to be added, such as reservoir water level [37], or ground temperature for freeze–thaw effects.

The systematic, often global, availability of satellite remote sensing data sources is a valuable addition to local (field) surveys and monitoring, where data availability is dependent on commissioning by local authorities. Furthermore, it allows for measurements in harsh environments that are not easily accessible. Here, we list potential information sources for each variable identified in Table 1.

### 2.1. Regional Topography

As gravity is the driving force behind any landslide, a slope is a requirement for landslide deformation. Local slope, aspect, and curvature are derived from globally available satellite digital elevation models, such as SRTM [38]; ALOS [39,40]; TanDEM-X DEM [41] or ASTER-GDEM [42]. Typically, the resolution of such products is 30–90 m. Regional products, mostly acquired from airborne platforms, often have a resolution of 0.5–10 m. A coarse resolution may hide small terrain features and will typically attenuate slope estimates [43,44,45], where on the contrary, a coarse elevation discretization may introduce false, sharp gradients [46].

The best elevation model for landslide characterisation is not necessarily the most accurate in the traditional sense. Errors in referenced specification documents are often listed as absolute errors, while the local error, relative to the direct vicinity, is much more relevant for the calculation of derivatives, such as slope. The quality of elevation models is debated in literature, often by intercomparison of different products [47,48,49,50,51,52] or comparison to a different measurement, such as GPS [53] or levelling [50]. Slopes, which are dominant in mountainous terrains, particularly have an effect on the accuracy of the elevation model [54]. Although acquisition dates vary, if topography is assumed to be stable, different sources can be combined. For example, MERIT [55], NASADEM [56], EarthEnv-DEM90 [57], and VFP [58] elevation models are fusion products, combining data from multiple elevation data products.

### 2.2. Regional Geology and Lithology

In the context of deep-seated landslide nowcasting, the geology can be considered static. However, not all lithologic types are equally susceptible to landslides. The OneGeology project serves as an integrator between various local maps and provides interfaces for public use to access these maps at a global scale. Global maps, such as the FAO-UNESCO “Digital Soil Map of the World” [59] or “ISLSCP II Global Gridded Soil Characteristics” [60], or dedicated maps, such as the lithological layer of the “International Hydrogeological Map of Europe” (IHME) [61], provide information on the uppermost water-bearing layer and matches the hydrological focus of landslide research. Resolutions are up to 100 m, which therefore covers mainly large-scale geologic features.

### 2.3. Hydro-Meteorology

Complete reviews of satellite precipitation data sets were given by Satgé et al. [62] and Beck et al. [63,64], each reviewing over 20 precipitation data sets of which half publish data within days. Both reviews conclude the best-performing data product is MSWEP, followed by IMERG. In the context of landslide nowcasting, only products with short availability are interesting: the product has to be available hours, or at maximum, a few days after the measurement, and should be open for integration. Products reported to have these properties are listed in Table 2. The average spatial resolution is approximately 12 km.

Antecedent water content, soil saturation, and pore pressure play key roles in landslide instability [25,28,65], and local soil moisture content can be a precursor of landslide instability [66,67]. Even though soil moisture measurements from space are still limited in resolution and depth, satellite soil moisture information may help constrain the nowcast [68].

A bucket or tank model can be used to estimate the ground water level from indirect measurements, such as precipitation, transpiration estimates, and run-off measurements [69,70,71,72]. Run-off of small streams cannot be measured by satellites, and requires in situ or close-range measurements, while the other parameters can be estimated [73]. This approximation is never perfect, but allows for an indirect estimate of groundwater from surface processes. The groundwater level is then used to estimate the pore pressure, which is related to the stability of the landslide [69,74]. Furthermore, it filters the high-frequency precipitation signal to low-frequency changes in groundwater.

### 2.4. Land Use

Land use has an influence on the infiltration of precipitation and the evapotranspiration loss of water, and therefore, is of influence to the hydrological cycle. Furthermore, artificial slopes are an alteration of the natural balance and are more susceptible to landslides [75]. Therefore, a combination of vegetation and topographic maps is required to assess the influence on the hydrological cycle.

OpenStreetMap provides a global coverage of the human presence in a unified way in large parts of the world on the scale of individual roads [76]. The large-scale CORINE Land Cover (CLC) map provides information on both anthropogenic settlements as well as crude vegetation information at 100 m resolution [77]. The JAXA ALOS forest map [78], as well as the ‘Global Forest Change’ product by Hansen et al. [79] focuses on forest cover only. Even larger-scale information on unmapped human settlements can be obtained from satellite observations of night light [80,81].

Unfortunately, land use information extracted from maps is limited by the update frequency. CORINE, for example, is updated every six years. Information extracted directly from satellite imagery is less prone to such delay, and enables the detection of land use changes and associated changes in the water balance. Satellite imagery can be used directly to monitor for extreme changes, such as forest fires or building activity, on a more frequent basis.

### 2.5. Displacement

Sources of deformation measurements are numerous, but often local. Inclinometers, total station measurements, and GNSS surveys provide on-site information on the progressive deformation of the landslide. The aforementioned solutions require access to the landslide to mount the sensors or benchmarks. Alternative solutions include ground-based InSAR (GB-InSAR) [82,83,84] and LiDAR surveys ([85,86,87], reviewed by [88]), that require no access to the landslide itself, but can be operated from anywhere with direct visibility on the landslide. Therefore, the system can be used as emergency intervention too [89]. However, especially continuous surveys require installation on-site. Measurements can only begin after installation, after sliding behaviour has been detected. Campaign-based measurements provide a limited temporal resolution, but may be operated without a fixed set-up on site [87].

Satellites are the ideal means for regional repeat surveys without access to the landslide or its vicinity. The importance of satellite-based Interferometric Synthetic Aperature Radar (InSAR) for both local and regional landslide deformation assessments is widely recognized [90,91], and Intrieri et al. [5] claim: “if InSAR monitoring had been active over this region, an early warning of imminent failure could have been given”. Especially for slow-moving landslides, the accurate tracking of ‘persistent scatterers’ (PS-InSAR), and often buildings or rock faces, provides opportunities for long-term deformation monitoring. Furthermore, the power of retrospective studies on previously acquired data is a big advantage over local monitoring solutions. However, constant features, such as clear rock faces or buildings should be present on the landslide. Moreover, the direction of sliding should not be parallel to the direction of flight of the satellite, typically north–south. Deformation in this direction will not be visible in the line of sight of the radar sensor, perpendicular to the direction of flight. Nevertheless, even with limited presence of such features, a combination with campaign-based surveys will still densify the deformation time-series [92,93,94].

## 3. Machine Learning and Data Assimilation

The integration methods discussed here combine different quantities from different measurements into a single, different quantity—deformation rate. A clear distinction can be made between either physical or statistical algorithms and qualitative or quantitative assessments. Physical modelling relies on expert knowledge of the processes in the landslide and is built on the evaluation of predefined sets of rules [18]. Statistical methods are based on the assumption that landslides are more likely to occur in circumstances that led to landslides earlier [36].

The assessment is either qualitative—such as low, moderate, or high hazard—or a quantitative output, such as deformation rate. The different assessment methods have been highlighted and illustrated in Table 3. The desired output for our approach is a deformation rate nowcast, estimated based on conditioning and triggering factors, as mentioned in Table 1. However, the existing, successful implementation of qualitative hazard nowcasting is a starting point for quantitative analysis. From there, we explore the possibilities of various quantitative algorithms with increasing computational complexity.

### 3.1. Hazard Nowcasting

Kirschbaum and Stanley [95] set an example for large-scale hazard nowcasting, and showed that simple rules can provide a qualitative landslide hazard nowcast. Their nowcast has global coverage and is updated every 30 minutes at a kilometer resolution based on satellite data. Their approach is to estimate susceptibility first, signaling a landslide hazard when thresholds on antecedent precipitation are exceeded in areas of high susceptibility. A similar method is used by Posner and Georgakakos [96], based on soil moisture instead of precipitation. These systems do not estimate the system state, and there is no nowcasting of deformation, as they are a nowcast of susceptibility instead.

Landslide hazard nowcasting and early warning systems are typically trained and tested on inventories of the time and place of historic, catastrophic landslides. These landslide inventories are often event inventories, listing collapse events rather than landslides experiencing continuous and slow deformation. Examples of inventories are the ‘Global Landslide Catalog’ [97], ‘ELS-DAT’ [2], and the ‘Global Fatal Landslide Database’ [1,98]. A system focused on continuous deformation patterns cannot be trained on the events in such inventories. Although shallow landslides are the primary focus of most large-scale inventories, some local inventories of deep-seated landslides exist [99,100], or can be deduced from deformation patterns [101,102,103]. Local inventories are still valuable to selectively activate nowcasting systems in areas with active landslides. While historic information could provide insight in the conditions leading up to the event, a catastrophic event will change the dynamics of the slope and previous dynamics may no longer be valid [36].

### 3.2. Deformation Nowcasting

A more complex approach is to estimate the system state, including the deformation rate, either by geomechanical modelling or based on statistics of historical deformation. This last approach of estimating the system state will be the focus of the methods mentioned here. Typically, solutions strive for the simplest model with the smallest possible error in the prediction of cumulative deformation or deformation rate. While more complex models are more likely to fit the data, they introduce the risk of overfitting the model to the data, thereby reducing the predictive power.

After model selection, as discussed in the following subsections, the process is typically subdivided into three steps: data preprocessing; training or optimisation, and application. During preprocessing, all variables are brought to the same reference frame. Furthermore, preprocessing of the input variables can be used to enhance the information content of the input, such as by dissecting the signal into various sub-signals first [113]. The training or optimisation phase is a computationally intensive phase, where the model parameters are optimised such that the model approaches the deformation process best. Many combinations of model and preprocessing and training methods are possible, and final selection may require multiple models to be tested [107]. Finally, during application, the tuned model is run over incoming data to predict the deformation of the landslide.

Intrieri et al. [14] reviewed a large number of data integration methods, and concluded that no ‘best’ model could be identified due to the lack of comparable case studies between models. The Baishuihe landslide, at the shores of the Three Georges Reservoir, China, offers some possibilities for comparison, as multiple methods have been tested on this landslide by various authors (see Table 4). However, the influence of the reservoir water level on the landslide stability, not commonly present elsewhere, cannot be neglected and conclusions are therefore not easily transferable to other landslides.

#### 3.2.1. Direct Relation Precipitation–Deformation

Traditional models, summarised by Bernardie et al. [116], rely on a direct relation between precipitation and deformation. Various models exist for this relation, where the parameters such as time lag are determined by optimisation on historical records. However, separate modelling of the hydro-meteorological conditions is required, as only effective precipitation can be used in the model. Support Vector Regression (SVR) is a data-driven equivalent of a direct relation, while Bossi and Marcato [105] found a direct relation with river discharge. However, a model with a direct relation between precipitation and displacement does not account for changing soil conditions and associated infiltration dynamics.

#### 3.2.2. Division of the Variable Space

Models such as Support Vector Machines (SVM), either with linear or non-linear models, subdivide the variable space in different combinations of conditioning factors. To find the optimal parameters for the model, an optimisation method is applied, such as Particle Swarm Optimization (PSO), Grid Search (GS) or Genetic Algorithm (GA), all applied by Miao et al. [107]. All optimisation parameters strive to use the change in output of each consecutive model state to further reduce the errors in the least possible iterations. Decision trees and Random Forest (RF) classifiers provide a similar (non-linear) subdivision of the input variables. All these models are insensitive to time series, although additional copies of input variables with a time lag may be added.

With slow-moving landslides, the deformation signal is small. Therefore, the absolute error of any deformation rate nowcast is likely to be small as well, and consequently difficult to compare to deformation rates modelled using physically-based models. Furthermore, when training (optimising) such models on a regional scale, there is a risk of introducing a bias towards the abundant stable, non-landslide cases. Thus, one has to provide both a balanced training sample, as well as an error metric (loss function) suitable for such small differences.

#### 3.2.3. Artificial Neural Networks

Powerful alternatives are Neural Networks and related technologies, which are not applied to classify the deformation behaviour, but to transform the input variables to a deformation estimate using a non-linear transformation. Such systems can be made aware of time and the spatial relationship between neighbouring areas and are capable of detecting relations unnoticed by experts. In addition, such systems may estimate other variables in the process, such as a groundwater change [33].

As the optimisation is based on statistics only, most machine learning algorithms are ‘unaware’ of the relations between system variables in space and time and are therefore unable to accurately asses the prediction error [117]. Additional rules may be implemented in the training processes, to validate the solution against physics or other rules. An example is the solution proposed by Karpatne et al. [118], the Physics Guided Neural Network (PGNN) that includes a physics-based model, integrating it into the error function. With the help of this model, state estimates with larger deviations from a realistic scenario are marked as less favourable.

A time-aware class of the Neural Networks are Recurrent Neural Networks (RNN), which operate on time series of variables and have some memory of previous states. Thanks to the ‘memory’ of those systems, previous conditions can be weighted, and when applicable, incorporated into the current state. An advanced implementation of this is Long Short-Term Memory (LSTM), a network capable of deciding whether a previous event is still applicable to the current state, retaining the memory—and if so, clearing it otherwise [119].

## 4. Discussion

The limited roots in physics of many machine learning algorithms provide new potential for the nowcasting of deep-seated landslides. With its origin in computer sciences, an understanding of real-world physics is not the primary focus of most machine learning algorithms and methods. This poses a challenge for the integration in early warning systems, that are traditionally an extension of expert judgement rather than an ‘expert’ in itself. Furthermore, future experts will combine the roles of landslide geologist and data scientist, combining information sources and bridging gaps in data availability.

### 4.1. Data Unification

Most machine learning techniques require all data to be in a consistent, monotonically increasing, spatio-temporal reference frame. Therefore, the resolution selection of the reference frame chosen has an influence on the outcome. Arnone et al. [120] concluded a 20 m spatial resolution was optimal, while Shirzadi et al. [121] concluded that a 10 m resolution was optimal. Both cases were higher than the resolution of the aforementioned satellite data products. Furthermore, re-projection between coordinate systems, as well as temporal interpolation, may introduce scaling of the original variable and a false perception of increased resolution.

Unified sampling is often required, with missing measurements blocking the process. Variables may need interpolation for features with lower spatial or temporal resolution, respecting the properties of the process underlying the variable, although higher resolutions in space and time may hide large-scale effects if analysis methods are not scaled appropriately with the increase in resolution. Moreover, in landslide nowcasting, the algorithm has to cope with missing data and the addition/update of historical data at a later stage.

Data cubes provide such unification of variables. The desired variables are preprocessed and spatiotemporally aggregated to a unified reference frame in space and time to facilitate data processing [122,123]. For such cubes, a multidimensional array of variable, time, *x* and *y* can be sliced in any direction (variable, time, or location) to disclose relations in time, space, or between variables. The cube can be generated on a project basis, with only the necessary variables [124,125], or be provided as ‘analysis-ready data’ by others [126,127]. However, for a time-critical application as nowcasting, these will have to be operational, ‘live’ data cubes.

### 4.2. Addition of Local Sensors

Local sensors may aid the interpretation of satellite products, and are a source of both calibration and validation for the satellite data products. For example, they can compensate for underestimated peak precipitation by satellite precipitation products due to spatial averaging [128] or validation of InSAR deformation analysis by GNSS sensors on the landslide (e.g., [129]). Local sensor data can be integrated in the process as well, especially when converted to the same reference frame as the satellite data products. Integration can be achieved either by mixing with the existing variables to increase local spatial or temporal resolution, or by rasterisation, creating a new variable from spatially interpolated sensor data. Furthermore, local observations allow for monitoring in geometries that are difficult to describe or observe from air or space, such as vertical walls [130,131].

Regional application of local sensors is not only feasible in the case of mass deployment of low-cost and low-maintenance sensors. Thomas et al. [66] showed that even a single soil moisture sensor may be representative of a larger region and better represent the soil moisture conditions than a satellite soil moisture product.

### 4.3. Addition of Physics

Physical constraints can be added at multiple stages of the process and bring the solution closer to the physical process. Simple physics, such as the tank model [69], may be used during variable pre-processing to amplify the information content of the variables. This integrates expert knowledge into the empirical system, unintentionally constraining the system to an assumed correlation. Including the same variable twice, once in a compound variable as well as independently, may over-represent the variable in the process.

During training of the algorithm, physics may be used to constrain the solutions to what is physically possible (e.g., landslides moving up-slope). This is implemented by the PGNN by penalizing solutions that are in conflict with such predefined rules [118]. A major drawback is that both solutions cannot compensate for errors in either the composite variables or constraints. Wrong assumptions will lead to sub-optimal training and predictive power.

### 4.4. Early Warning Systems

The slow deformation behaviour of deep-seated landslides will not often prompt situations of immediate collapse or those that are life-threatening. However, warnings for strong acceleration, and associated risk for building and infrastructure damage, could be raised from a monitoring system. Early warning systems, solely based on (local) deformation measurements, have to detect precursory acceleration before a warning can be raised. Meanwhile, infrastructure and buildings on the slope are already undergoing increased stresses.

With satellite deformation tracking only, multiple acquisitions are necessary to trigger an alarm [5,132]. Current algorithms require at least 4–5 radar observations indicating acceleration to come to an early warning with a reasonably low false alarm rate [5]. For example, given the 6-day revisiting time of the ESA Sentinel 1 satellite mission, this is a lead time of 24 days. Therefore, integration with other, more frequent measurements is necessary to come to an early warning and accelerate the detection of deformation anomalies. Moreover, a nowcasting system, including both driving and resulting factors, is less sensitive to the timeliness of the inputs and more suited for incorporation in an early warning system. However, special attention should be given to verify the performance for early warning of catastrophic accelerations of deep-seated landslides due to the rarity of such events.

Integration of the different data sources is an ongoing challenge faced by many remote sensing projects. The methods can be subdivided into traditional, statistical, or signal processing methods and artificial intelligence. The traditional methods have a mathematically defined behaviour, where the latter have proven to be very effective in recent applications, but are considered *blackboxes*.

The nowcasting system has to value known, historical information with respect to the current state. Traditional methods have limited flexibility in this respect. Possible pitfalls of the flexibility of machine learning methods are overfitting on previous deformation and unrealistic predictions due to the lack of physical constraints.

Unfortunately, it is practically impossible to guarantee that the more complex machine learning algorithms will always yield the desired warning, as it is impossible to simulate all time series and the non-linear behaviour does not allow for interpolation of the results. Such behaviour is undesirable in early warning systems, where there is a delicate balance in the perception of false alarms and missed detections [133]. Furthermore, it poses the question of how to present the new, uncertain results to the public—an early warning system is only complete with a communication framework to distribute the warnings raised [134].

By integrating the nowcast in the early warning system, alarms are now based on the interplay of variables, contrary to established single-variable intensity-duration thresholds. Furthermore, ensemble predictions, showing agreement or disagreement between ensemble members, can be used to warn against uncertain predictions. To increase predictive power, weather forecasts can be used to detect such problems beforehand [135]. Together, these provide opportunities for the implementation of an unconventional but trustworthy warning system.

The application of continuous learning—the continuous optimization of the model to the newly recorded data—allows for the adaption to changing conditions, integrating new situations not encountered in the initial training phase and learning from previously missed stabilization or acceleration. However, continuous learning may mask slow changes, falsely updating the ground state.

### 4.5. Risk Assessment and Reduction

The quantitative hazard estimate from the nowcasting system can be projected onto objects of socio-economic importance, such as infrastructure and housing. The initial hazard estimate is hereby upgraded to a risk estimate—listing the potential damage incurred by the nowcasted, accelerated deformation. Moreover, the processing priority can be guided by the objects at risk, processing areas of high importance first or more frequently, thus maintaining regional coverage at the reduced computational cost. Furthermore, the empirical relations derived from the training of the machine learning algorithm are a valuable resource for the planning of mitigation measures.

## 5. Conclusions

Instead of describing the exact dynamics of each landslide, machine learning may serve a similar purpose in local and regional nowcasting and early warning systems. The continuous, wide-area time series from satellite remote sensing offer a unique opportunity to monitor deformation and hydro-meteorological conditions of landslides on a local and regional scale. In this paper, we showed that there are satellite remote sensing products available that capture the major contributors to the landslide process as well as the continuous, slow deformation of deep-seated landslides themselves.

The limited frequency of deformation updates necessitates the integration of data from other, more frequent, sources to continuously estimating the current system state. Simple physics and proxy indicators may compensate for variables that cannot be observed directly from space. The different machine learning algorithms we listed have been demonstrated to be capable of processing the large data streams available to a nowcast of deformation on a local scale.

A satellite remote sensing landslide nowcasting system can be applied on demand, and has the potential to be applied globally, independent of terrain accessibility or local budget, and provides additional protection to those affected. However, integration in early warning systems on both a local and regional scale will require further refinement of the algorithms and a new approach to ‘live’, unified data integration.

## Figures and Tables

**Table 1:** Key variables in the landslide process that can be acquired from satellite observations.

Variable	Role	
Slope	Pre-disposing	Static
Geology	Pre-disposing	Static
Soil moisture	Causal	Dynamic
Precipitation	Trigger	Dynamic
Snow (melt)	Trigger	Dynamic
Land use	Causal	Dynamic
Deformation	Result	Dynamic

**Table 2:** Overview of precipitation data products. Adapted from Satgé et al. [62] and Beck et al. [63,64], showing only data sources with at least Near Real Time (NRT) coverage. A spatial resolution of 0.1° is approximately equal to 11×11 km at the equator, or 8×11 km at 45° N/S (e.g., Alps). Lag is the relative age of the latest available product.

Name	Spatial		Temporal		Lag	Note
	Resolution	Coverage	Resolution	Coverage		
CMORPH v1.0	0.07°	<60°	30 min	1998-NRT	1 day	
GDAS	∼0.25°	global	3 hourly	2015-NRT	6 days	
GSMaP-MVK	0.1°	<60°	hourly	2000-NRT	3 days	
GSMaP-NRT	0.1°	<60°	hourly	2008-NRT	4 h	
GSMaP-NOW	0.1°	<60°	30 min	2019-NRT	30 min	
GSMaP-RNC	0.1°	<60°	hourly	NRT only	−6 h	
IMERG v5/6	0.1°	<60°	30 min	2014-NRT	4 h	
MSWEP v2.2	0.1°	global	3 hourly	1979-NRT		Request only
PERSIANN	0.25°	<60°	hourly	2000-NRT	2 days	
PERSIANN-CCS	0.04°	<60°	hourly	2003-NRT	1 h	
TMPA 3B42RT v7	0.25°	<50°	3 hourly	2000-NRT	8 h	Obsolete
CPC Unified	0.5°	land	daily	1979-NRT	2 days	
ERA5T	0.25°	global	hourly	1979-NRT	5 days	

**Table 3:** The methods of landslide susceptibility or hazard assessment discussed, and their properties in time and type of analysis. (Map icon derived from [10]).

Method	Time Dependency	Outcome	
Susceptibility mapping	None, static	Qualitative	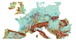
Hazard nowcasting	Dynamic	Qualitative	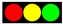
Deformation nowcasting	Dynamic	Quantitative	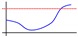

**Table 4:** Examples of different integration methods, linking hydro-meteorological conditions to deformation time series, and associated case studies. Where applicable, the reference methods used in the paper are listed in brackets. Relevant abbreviations are expanded in the text.

	Case Study	Observed Driving Forces	Deform. meas.	Method (Reference Methods)
Xie et al. [104]	Laowuji, China	Rainfall, toe excavation	Total Station	LSTM
Bossi and Marcato [105]	Passo della Morte, Italy	Rainfall, groundwater	Inclinometer	Linear regression
Yang et al. [106]	Baishuihe & Bazimen, China	Rainfall, reservoir level	GNSS	LSTM
Miao et al. [107]	Baishuihe, China	Rainfall, reservoir level	GNSS, inclinometer	GA-SVR, GS-SVR, PSO-SVR
Li et al. [37]	Baishuihe, China	Rainfall, reservoir level	GNSS	LASSO-ELM, Copula (ELM, SVM, RF, kNN)
Logar et al. [108]	Ventor, United Kingdom	Rainfall	Crackmeter	ANN
Krkač et al. [33]	Kostanjek, Croatia	Groundwater (change), season	GNSS	RF
Zhou et al. [109]	Bazimen, China	Rainfall, reservoir level	GNSS	PSO-SVM (GA-SVM, GS-SVM, BPNN)
Cao et al. [110]	Baijiabao, China	Rainfall, groundwater, reservoir level	GNSS	ELM (SVM)
Lian et al. [111]	Baishuihe & Bazimen, China	Rainfall, reservoir level	GNSS	LSSVM, ELM, combination
Chen and Zeng [112]	Baishuihe, China	None	GNSS	BPNN
Du et al. [31]	Baishuihe & Bazimen, China	Rainfall, reservoir level	GNSS, inclinometer	BPNN
Lian et al. [113]	Buishuihe, China	None	GNSS	EEMD-ELM, M-EEMD-ELM (ANN, BPNN, RBFNN, SVR, ELM)
Corominas et al. [114]	Vallcebre, Spain	Groundwater	Extensometers	Physics
Neaupane and Achet [115]	Okharpauwa, Nepal	Rainfall, groundwater	Autoextensometer	BPNN
